# Overall Survival and Associations of Insurance Status Among Hispanic Men With High-Risk Prostate Cancer

**DOI:** 10.7759/cureus.45723

**Published:** 2023-09-21

**Authors:** Zachariah Taylor, Stephanie Kjelstrom, Meghan Buckley, David Cahn

**Affiliations:** 1 Urology, Main Line Health, Philadelphia, USA; 2 Statistics, Lankenau Institute for Medical Research, Wynnewood, USA; 3 Urology, MidLantic Urology, Media, USA

**Keywords:** cancer epidemiology, large-database, overall survival (os), hispanic population, prostate cancer (pca)

## Abstract

Objectives

Our objectives were to (1) determine the association between ethnicity and high-risk prostate cancer (PCa) survival and (2) determine whether this association is modified by insurance status.

Methods

We performed a retrospective review of the National Cancer Database (NCDB) from 2004 to 2017 of non-Hispanic White (NHW), Hispanic White (HW), or Black men with high-risk PCa. A multivariate Cox regression model was built to test the association between overall survival (OS) and race/ethnicity, insurance status, and their interaction, controlling for various socioeconomic and disease-specific variables.

Results

A total of 94,708 men with high-risk PCa were included in the analysis. Both HW and Black men had lower socioeconomic status characteristics and lower rates of private insurance. Race/ethnicity was significantly associated with OS in the adjusted analysis. Only Medicare demonstrated significantly worse OS. NHW (covariate-adjusted hazard ratio (aHR): 1.83, 95% CI: 1.45-2.32) and Black (aHR: 1.71, 05% CI: 1.34-2.19) men demonstrated significantly worse survival when compared to HW men. Subgroup analysis demonstrated significant differences occurring among HW men with private insurance/managed care when compared to those not insured, Medicaid, Medicare, and other government insurance types.

Conclusion

Despite socioeconomic and demographic disadvantages, HW men demonstrate improved OS compared to NHW men. Furthermore, HW men demonstrated improved OS compared to NHW men within nearly each insurance status type. This finding is likely the result of a complex multifactorial web and as such serves as an interesting hypothesis-generating study.

## Introduction

Prostate cancer (PCa) is the most common malignancy in men, with an estimated incidence of 268,490 new cases and 34,500 deaths in the United States (US) in 2022 [1]. High-risk PCa has recently been shown to be becoming more common with the proportional rates of high-risk PCa increasing from 11.8% in 2014 to 20.4% in 2016 [2]. Notably, however, distant-stage PCa increased temporally, where Siegel et al. demonstrated that in 2017, only 70% of PCa were localized, compared to 78% in 2003 [3]. This is likely due to a combination of improved sensitivities of diagnostic modalities such as positron emission tomography [4], more common utilization of prostate magnetic resonance imaging [5], and possibly the downstream effect of the 2012 US Preventive Services Task Force Grade D designation of prostate-specific antigen (PSA) screening [6]. Both radical prostatectomy (RP), as well as external beam radiation therapy with or without androgen deprivation therapy (ADT), have shown to be effective frontline therapies for men with non-metastatic, locally advanced, or high-risk PCa, whereas combination systemic treatment with ADT, oncolytics, radiotherapy, chemotherapy, immunotherapy, clinical trials, or others, is indicated for those with metastatic disease [7].

Previous studies have shown that access to multidisciplinary care at high-volume tertiary care academic centers is associated with improved survival [8]. On the contrary, barriers to care, such as insurance status, have been associated with worse survival outcomes, where previous studies have shown that uninsured men experienced 103% higher overall and 61% PCa-specific mortality compared to men with Medicaid or private insurance [9]. Furthermore, men with government-funded insurance such as Medicaid or Medicare have also demonstrated worse overall survival (OS) than men with private insurance [10].

Private insurance rates have been demonstrated to be higher among non-Hispanic White (NHW) men. Previous studies using the Surveillance, Epidemiology, and End Results (SEER) database demonstrated that 86% of NHW individuals from age 18-64 diagnosed with cancer were privately insured versus 72% of Black and only 68% of Hispanic White (HW) [10]. While the association between insurance status and race has been studied in NHW and Black men, it has not been thoroughly investigated in HW men [11]. In this study, our objectives were to (1) determine the association between ethnicity and high-risk PCa survival and (2) determine whether this association is modified by insurance status.

## Materials and methods

This study was a retrospective review of the National Cancer Database (NCDB), established in 1989 as a jointly sponsored database between the American College of Surgeons and the American Cancer Society to serve as a comprehensive clinical surveillance resource. The NCDB sources hospital registry data from more than 1,500 commission-on-cancer-accredited facilities and contains more than 70% of newly diagnosed cancer cases nationwide [12].

**Figure 1:**
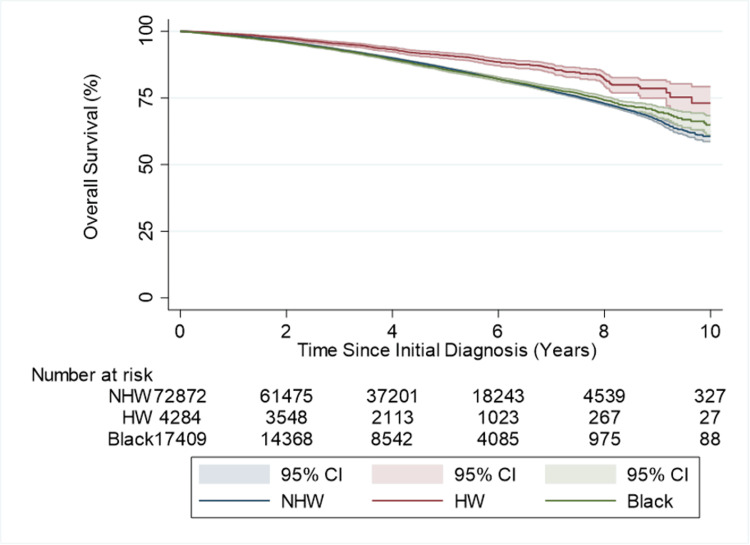
Unadjusted OS of HW, NHW, and Black men NHW: non-Hispanic White, HW: Hispanic White; 95% CI=95% CI

Patients with PCa were identified in the NCDB based upon the International Classification of Diseases for Oncology third edition (ICD-O-3) site code C61.9. The study cohort was confined to individuals aged ≥18 diagnosed with high-risk PCa between 2004 and 2017. Patients were characterized as having high-risk disease if they had clinical stage T3-T4, a PSA level greater than 20 ng/mL, or a Gleason score of 8-10. Patients with metastatic disease, non-adenocarcinoma histology, missing survival data, missing PSA value, missing Gleason score, missing T-stage, or missing demographic information were excluded from the analysis, as seen in Figure 1.

The following patient demographics, cancer characteristics, and treatment modalities were recorded for each patient in the NCDB: age at diagnosis (years), primary insurance at diagnosis (not insured, private insurance/managed care, Medicaid, Medicare, other government), ethnicity (not of Spanish/Hispanic origin, Spanish/Hispanic origin), percentage of residents in the patient’s zip code without a high school degree (as estimated in the 2000 US Census: ≥29%, 20-28.9%, 14-19.9%, <14%), median household income (as estimated in the 2000 US Census: <$30,000, $30,000-$34,999, $35,000-$45,999, ≥$46,000), patient location (as measured by the US Department of Agriculture’s Economic Research Service: urban, rural, metropolitan), Charlson-Deyo score (0, 1, 2, or more), Gleason score, PSA score, treatment type (radiation only, radical or total prostatectomy, radiation, and prostatectomy), whether the patient received chemotherapy and/or hormone therapy, facility type (community cancer program, comprehensive community cancer program, academic/research program, integrated network care program), and facility location based the reporting facility’s US Census Division.

The primary outcome, OS, was defined as the time (years) between the date of initial PCa diagnosis and the date of last contact or death, whichever occurred first. Since OS may be impacted by the presence of other cancers, we only included men whose PCa was their only lifetime malignancy. Additional exclusion criteria included non-adenocarcinoma histology, unknown time from diagnosis to start of radiation and/or radical or total prostatectomy, unknown if chemotherapy or hormone therapy was administered, unknown time from diagnosis to start of hormone or chemotherapy, and missing data. The full list of exclusion criteria can be found in Figure 1.

Patients were classified into one of three mutually exclusive race/ethnic groups for analytic purposes: NHW, HW, and Black.

Continuous data are presented as mean (SD) or median (IQR), and categorical data is presented as frequency (percentage). The normality of continuous distributions was assessed using histograms.

Patient demographics at diagnosis, cancer characteristics, treatment types, and facility types were compared across NHW, HW, and Black patients using the Chi-square test of independence (categorical distributions), ANOVA (normally distributed continuous distributions), and the Kruskal-Wallis test (non-normally distributed continuous distributions). If significant, then follow-up pairwise comparisons were made using the Chi-square test of independence, Bonferroni-corrected two-sample t-test, and the Wilcoxon rank-sum test, respectively. The same methods were also used to compare patients who were included vs. excluded from the analytic cohort.

Univariable Cox proportional hazard models were built to test the associations between patient demographics at diagnosis, cancer characteristics, treatment types, facility types, and OS. HR and 95% CI are presented. Survival distributions were estimated and graphed using Kaplan-Meier curves, and curves were compared using the log-rank test.

A multivariable Cox proportional hazards model was used to test the hypothesis that insurance type modified the effect of race/ethnicity and OS. This model included the main effects of race/ethnicity, primary insurance at diagnosis, and the interaction between race/ethnicity and primary insurance at diagnosis. The model also controlled for the following potential confounding variables: age at diagnosis (years), percentage without a high school degree, median household income, patient location (urban or rural vs. metropolitan), facility type, facility location (collapsed into the following categories due to sparse data: New England/Middle Atlantic/South Atlantic, East/West Central, Mountain, and Pacific), Charleson-Deyo score, Gleason score, PSA, days from diagnosis to start of radiation, days from diagnosis to start of chemotherapy, days from diagnosis to most definitive surgery, and days from diagnosis to hormone therapy. The proportional hazard assumption was tested for each covariate as well as globally.

A priori, it was decided that subgroup analyses would be performed for exploratory purposes, regardless of the significance of the interaction between race/ethnicity and primary insurance at diagnosis. The subgroup analyses examined the association between race/ethnicity and OS within each type of primary insurance. No p-value adjustments were made for the subgroup analyses. Covariate-adjusted hazard ratios (aHR) and 95% CIs are presented.

All tests were two-sided and statistical significance was assessed at the 5% level. Analyses were performed in Stata/MP 15.1 (StataCorp LP., Texas, USA).

## Results

A total of 1,623,100 men between the ages of 18 and 90 were identified as having PCa in the NCDB database between 2004 and 2017. Of these, 94,708 men met the inclusion criteria and remained eligible for analysis.

The distribution of patient demographics at diagnosis, cancer characteristics, treatment types, and facility types and locations are displayed in Table 1. Among the 94,708 men in the analytic cohort, 72,965 (77.04%) were NHW, 4,299 (4.53%) were HW, and 17,444 (18.46%) were Black. Statistically significant differences were identified across the race/ethnicity groups in insurance status, Gleason score, PSA, and treatment type, among others.

**Table 1:** Distribution of patient demographics and clinical characteristics by race and ethnicity SD: standard deviation, IQR: interquartile range, PSA: prostate-specific antigen ^1^P-value compares distributions across all three race and ethnicity groups ^2^Based on the 2016 American Community Survey, spanning the years 2012-2016 ^3^Based on the 2000 US Census

		White		Black		
		Non-Hispanic/Spanish	Hispanic/Spanish			Total
Patient, cancer, and facility characteristics		(n=54,764)	(n=3,011)	(n=9,463)	p-value^1^	(n=67,238)
Age at diagnosis (years), mean (SD)		63.9 (7.5)	63.0 (7.9)	61.0 (7.5)	<0.001	63.4 (7.6)
Ethnicity, n (%)					---	
	Not of Spanish/Hispanic origin	54,764 (100.0)	---	9,330 (98.6)		64,094 (95.3)
	Spanish/Hispanic origin	---	3,011 (100.0)	133 (1.4)		3,144 (4.7)
Primary insurance at diagnosis, n (%)					<0.001	
	Not insured	696 (1.3)	219 (7.3)	449 (4.7)		1,364 (2.0)
	Private insurance/managed care	28,902 (52.8)	1,332 (44.2)	4,696 (49.6)		34,930 (52)
	Medicaid	1,057 (1.9)	330 (11.0)	960 (10.1)		2,347 (3.5)
	Medicare	23,090 (42.2)	1,092 (36.3)	3,044 (32.2)		27,226 (40.5)
	Other government	1,019 (1.9)	38 (1.3)	314 (3.3)		1,371 (2.0)
Percentage without high school degree, n (%)^2^					<0.001	
	≥17.6%	6,706 (12.3)	1,571 (52.2)	3,455 (36.5)		11,732 (17.5)
	10.9-17.5%	12,315 (22.5)	607 (20.2)	2,984 (31.5)		15,906 (23.7)
	6.3-10.8%	17,336 (31.7)	448 (14.9)	1,970 (20.8)		19,754 (29.4)
	<6.3%	18,407 (33.6)	385 (12.8)	1,054 (11.1)		19,846 (29.5)
Median household income quartile, n (%)^3^					<0.001	
		6,214 (11.4)	772 (25.6)	3,746 (39.6)		10,732 (16.0)
	$30,000-$34,999	11,274 (20.6)	690 (22.9)	2,025 (21.4)		13,989 (20.8)
	$35,000-$45,999	13,550 (24.7)	708 (23.5)	1,577 (16.7)		15,835 (23.6)
	≥$46,000	23,726 (43.3)	841 (27.9)	2,115 (22.4)		26,682 (39.7)
Patient location, n (%)					<0.001	
	Urban or rural	10,127 (18.5)	174 (5.8)	825 (8.7)		11,126 (16.6)
	Metropolitan	44,637 (81.5)	2,837 (94.2)	8,638 (91.3)		56,112 (83.5)
Charlson-Deyo score, n (%)					<0.001	
	0	44,521 (81.3)	2,377 (78.9)	7,148 (75.5)		54,046 (80.4)
	1	8,325 (15.2)	538 (17.9)	1,810 (19.1)		10,673 (15.9)
	2 or more	1,918 (3.5)	96 (3.2)	505 (5.3)		2,519 (3.8)
Analytic stage, n (%)					<0.001	
	III	43,980 (80.3)	2,262 (75.1)	7,110 (75.1)		53,352 (79.4)
	IV	10,784 (19.7)	749 (24.9)	2,353 (24.9)		13,886 (20.7)
Gleason score, median (IQR)		7 (7-8)	7 (7-8)	7 (7-8)	0.002	7 (7-8)
PSA (ng/ml), median (IQR)		7.5 (5.1-13.9)	9.6 (5.9-19.5)	10.4 (6.1-24.0)	<0.001	7.9 (5.2-15.1)
Treatment type, n (%)					<0.001	
	Radiation only	9,039 (16.5)	656 (21.8)	2,154 (22.8)		11,849 (17.6)
	Radical or total prostatectomy only	38,291 (69.9)	1,915 (63.6)	6,023 (63.7)		46,229 (68.8)
	Radiation + radical or total prostatectomy	7,434 (13.6)	440 (14.6)	1,286 (13.6)		9,160 (13.6)
Chemotherapy administered, n (%)		1,001 (1.8)	69 (2.3)	173 (1.8)	0.182	1,243 (1.9)
Hormone therapy administered, n (%)		14,890 (27.2)	980 (32.6)	2,948 (31.2)	<0.001	18,818 (28.0)
Facility type, n (%)					<0.001	
	Community cancer program	3,431 (6.3)	141 (4.7)	494 (5.2)		4,066 (6.1)
	Comprehensive community cancer program	20,555 (37.5)	985 (32.7)	2,928 (30.9)		24,468 (36.4)
	Academic/research program	23,811 (43.5)	1,580 (52.5)	4,842 (51.2)		30,233 (45)
	Integrated network cancer program	6,967 (12.7)	305 (10.1)	1,199 (12.7)		8,471 (12.6)
Facility location, n (%)					<0.001	
	New England	4,002 (7.3)	109 (3.6)	299 (3.2)		4,410 (6.6)
	Middle Atlantic	7,942 (14.5)	445 (14.8)	1,536 (16.2)		9,923 (14.8)
	South Atlantic	9,903 (18.1)	502 (16.7)	2,882 (30.5)		13,287 (19.8)
	East North Central	10,771 (19.7)	280 (9.3)	1,695 (17.9)		12,746 (19)
	East South Central	4,018 (7.3)	17 (0.6)	981 (10.4)		5,016 (7.5)
	West North Central	5,756 (10.5)	72 (2.4)	409 (4.3)		6,237 (9.3)
	West South Central	3,121 (5.7)	511 (17)	1,076 (11.4)		4,708 (7)
	Mountain	2,608 (4.8)	204 (6.8)	69 (0.7)		2,881 (4.3)
	Pacific	6,643 (12.1)	871 (28.9)	516 (5.5)		8,030 (11.9)

HW patients were the most likely to be uninsured at the time of diagnosis (7.7% vs. 1.2% in HW and 5.0% in Black) and were also the less likely to have private insurance/managed care (35.5% vs. 38.8% in NHW and 38.4% in Black). HW patients were also the least likely to have a high school degree and demonstrated lower average income. There were no differences in Gleason score across the three groups. HW demonstrated a higher PSA at diagnosis than NHW (14.2 ng/mL vs. 10.5 ng/mL); however, both groups had a lower median PSA than Black men (20.2 ng/mL). See Table 1 for the full demographic and clinical differences between HW, NHW, and Black men in the study. In the unadjusted analyses, NHW demonstrated worse OS when compared to HW (HR: 1.58, 95% CI: 1.42-1.76) as did Black men (HR: 1.57, 95% CI: 1.40-1.76; p=<0.001), as seen in Figure 1. Patients who were not insured or had Medicaid, Medicare, or other government insurance all had significantly worse OS compared to patients with private insurance/managed care (HR ranged from 1.71 (95% CI: 1.51-1.94; p=<0.001) (not insured) to 2.49 (95% CI: 2.39-2.60; p=<0.001) (Medicare)).

Race/ethnicity was still significantly associated with OS in the multivariable model with HW (aHR: 0.73, 95% CI: 0.58-0.92, p<0.008) demonstrating improved OS when compared to NHW. There was no statistically significant difference between Black men and NHW (aHR: 0.90, 95% CI: 0.80-1.02). Uninsured (aHR: 1.63, 95% CI: 1.37-1.95), Medicaid (aHR: 1.67, 95% CI: 1.43-1.94), and Medicare (aHR: 1.21, 95% CI: 1.13-1.29) all demonstrated worse OS when compared to private insurance. No significant interaction effect between race/ethnicity and primary insurance was present (Table 2). That is, the effect of race/ethnicity on OS did not differ by primary insurance type.

**Table 2:** Multivariable proportional hazard Cox regression model aHR: covariate-adjusted hazard ratio, CI: confidence interval, LCL/UCL: lower/upper confidence limit, HW: Hispanic White, NHW: non-Hispanic White The model controls for age at diagnosis, percentage without a high school degree, median household income, patient location (urban or rural vs. metropolitan), facility type, facility location, Charleson-Deyo score, surgery, radiation, chemotherapy administered, hormone therapy, time from diagnosis to radiation, time from diagnosis to chemotherapy, time from diagnosis to surgery, and time from diagnosis to hormone therapy

	NHW	Reference	---	---	---
	HW	0.73	0.58	0.92	0.008
	Black	0.9	0.8	1.02	0.091
Primary insurance at diagnosis					<0.001
	Not insured	1.63	1.37	1.95	<0.001
	Private insurance/managed care	Reference	---	---	---
	Medicaid	1.67	1.43	1.94	<0.001
	Medicare	1.21	1.13	1.29	<0.001
	Other government	1.1	0.91	1.34	0.319
Race/ethnicity by insurance interaction (ref = NHW, private insurance/managed care)					0.149
	HW, not insured	0.99	0.63	1.56	0.981
	HW, Medicaid	1.05	0.71	1.55	0.81
	HW, Medicare	0.86	0.64	1.16	0.334
	HW, other government	0.42	0.1	1.72	0.227
	Black, not insured	0.77	0.57	1.04	0.086
	Black, Medicaid	0.96	0.75	1.22	0.715
	Black, Medicare	1.12	0.96	1.3	0.146
	Black, other government	1.07	0.72	1.6	0.735

Further, NHW patients had a worse OS compared to HW patients in private insurance (aHR: 1.37: 95% CI: 1.09-1.72) and Medicare groups (aHR: 1.58, 95% CI: 1.31-1.91). However, no statistically significant difference was observed in the uninsured or Medicaid groups (Table 3).

**Table 3:** Subgroup analyses of race/ethnicity and insurance type aHR: covariate-adjusted hazard ratio, CI: confidence interval, LCL/UCL: lower/upper confidence limit, NHW: non-Hispanic White, HW: Hispanic White

	Not insured				Private insurance/managed care			
		95% CI				95% CI		
	aHR	LCL	UCL	p-value	aHR	LCL	UCL	p-value
NHW vs. HW (reference)	1.37	0.93	2.04	0.114	1.37	1.09	1.72	0.008
Black vs. HW (reference)	0.96	0.63	1.45	0.835	1.24	0.96	1.58	0.096
Black vs. NHW (reference)	0.70	0.53	0.92	0.010	0.90	0.80	1.02	0.091
	Medicaid				Medicare			
		95% CI				95% CI		
	aHR	LCL	UCL	p-value	aHR	LCL	UCL	p-value
NHW vs. HW (reference)	1.30	0.94	1.80	0.108	1.58	1.31	1.91	<0.001
Black vs. HW (reference)	1.13	0.81	1.56	0.473	1.59	1.30	1.96	<0.001
Black vs. NHW (reference)	0.86	0.70	1.07	0.183	1.01	0.91	1.12	0.851

## Discussion

Our study demonstrated that with an adjustment for all socioeconomic and clinical factors, HW men with high-risk PCa had a lower risk of death when compared to NHW. Insurance was also associated with OS, where uninsured, Medicaid, and Medicare all demonstrated worse OS when compared to privately insured patients. Further subgroup analysis demonstrated that only Medicare was associated with worse OS within the HW group. Our study expands upon previously published data that demonstrated the presence of a Hispanic paradox in urologic malignancies, now more specifically in patients with high-risk PCa [13].

The underlying reason for the improved OS in the HW population remains unclear. Given worse socioeconomic factors, such as education and average income, it would be expected that the HW population would have worse OS when compared to the NHW group. Few theories exist as to why the Hispanic paradox has been observed.

The healthy migrant hypothesis supports the presence of a Hispanic paradox, suggesting those who immigrate to a different country are typically younger and healthier, selecting a population with better health and survival outcomes [14]. Limited data exist describing the survival differences between first- and second-generation Hispanics. However, US-born Latinos may have worse survival than their foreign-born counterparts, as previous studies have demonstrated mortality rates for US-born Latinos are approaching that of NHW [15].

While the Hispanic paradox has been described in other non-urologic conditions such as cirrhosis [16] and non-small cell lung cancers [17], it has not been universally observed in all malignancies. Previous studies on bladder cancer and retroperitoneal sarcomas have demonstrated no significant difference between HW and NHW regarding OS [18,19]. Furthermore, worse survival outcomes have been described for HW men with colorectal cancers [20] or with testicular cancers, residing in the US-Mexico border region [21].

Socioeconomic status, as well as insurance status, is a well-known predictor of health outcomes [9]. Our study demonstrated that private insurance has an unsurprising strong survival benefit over other insurance types in the NHW and Black groups. However, Medicaid insurance demonstrated no survival benefit when compared to uninsured in any group. The benefit of private insurance has been well established with survival advantages attributed to factors such as availability of transportation, ability to take time off work for medical appointments, and greater ease in finding a provider who will accept their insurance [22]. While both Medicaid and uninsured individuals likely do not benefit from many of the survival benefits associated with private insurance, it is unclear why Medicaid insurance does not offer survival benefits over uninsured individuals. One possible cause for no improvement in survival may be the disability requirements necessary for Medicaid enrollment, thus selecting a higher-risk population [23].

It has been suggested that receipt of appropriate optimal and guideline-based cancer care may be easier to obtain with private insurance [9]. Lichtensztajin et al. demonstrated that men with high-risk localized PCa were less likely to receive guideline-concordant care when compared to the NHW population in their study [24]. They theorized that the treatment disparity could be accounted for by socio-demographic barriers to care and potentially addressed through targeted interventions aimed at these barriers to care [24]. While our study supports that HW has worse socioeconomic factors, it is interesting that HW men have improved survival despite possible barriers to care.

Previous studies have demonstrated that Black men demonstrate higher PSA at diagnosis [25], more advanced stage [26], and worse survival when compared to NHW [3]. These findings have led to concern for biological and environmental causes of worse PCa survival. Recent studies have refuted the idea of a biologically distinct form of aggressive PCa in Black men [27]. Dess et al. recently demonstrated that when Black men have similar access to care and receive standardized treatment, they have comparable stage-for-stage PCa-specific mortality in comparison to NHW [28]. This data suggests that socioeconomic and environmental factors, and not tumor biology, cause disparate outcomes in this population [28]. This gives reason to suspect that the improved survival outcomes observed in HW men may also be environmental in nature.

This study has several limitations that are important to consider when interpreting the results. First, this was a retrospective study, which included inherent biases that were attempted to be controlled with the design and modeling of the study. Further, this study was limited to the variables that are aggregated by the NCDB. This includes data such as country of origin and limited data regarding reasons for not receiving treatment. NCDB does not record cancer-specific mortality, leading us to use OS as our outcome. Insurance data is obtained from the primary payer at the time of diagnosis. However, if the payer at the time of diagnosis is not known, the payer at the time of treatment is recorded which allows for the possibility of misclassification of some men in this study. Further, it is not known how many of the men who were diagnosed without insurance went on to obtain insurance after their diagnosis which would affect their ultimate treatment course. Finally, because this is a retrospective study, patients were not randomized to treatment modalities, and selection bias may have influenced the distribution of patients between treatment methods.

## Conclusions

In this study, we demonstrated that HW had improved OS than NHW men despite socioeconomic factors that generally are typically associated with worse outcomes. Demographic variables in our study demonstrated that HW and Black men had lower socioeconomic status than NHW men. While these factors are certainly affecting the clinical course for these men diagnosed with PCa, it is interesting that HW men show improved OS despite demographic characteristics associated with worse outcomes. Further, there was no association between insurance status and race/ethnicity found. This finding is likely the result of a complex multifactorial web, which serves as interesting hypothesis-generating data. Further studies are needed to better understand the outcomes of Hispanic men with high-risk PCa.
